# Opportunistic Allocation of Resources for Smart Metering Considering Fixed and Random Wireless Channels

**DOI:** 10.3390/s25082570

**Published:** 2025-04-18

**Authors:** Christian Jara, Juan Inga, Esteban Inga

**Affiliations:** 1 Master of Electricity Program (MEL), Department of Master’s Degree in Electricity, Universidad Politécnica Salesiana, Cuenca EC010102, Ecuador; 2 Telecommunications and Telematic Research Group (GITEL), Universidad Politécnica Salesiana, Cuenca EC010102, Ecuador; 3 Smart Grids Research Group (GIREI), Universidad Politécnica Salesiana, Cuenca EC010102, Ecuador

**Keywords:** resource optimization, wireless channels, cellular networks, smart meters, cognitive mobile virtual network operator, channel allocation, Hungarian algorithm, matching, congestion management, data transmission

## Abstract

This paper presents an optimization model for wireless channel allocation in cellular networks, specifically designed for the transmission of smart meter (SM) data through a mobile virtual network operator (MVNO). The model efficiently allocates transmission channels, minimizing smart grid (SG) costs. The MVNO manages fixed and random channels through a shared access scheme, optimizing meter connectivity. Channel allocation is based on a Markovian approach and optimized through the Hungarian algorithm that minimizes the weight in a bipartite network between meters and channels. In addition, cumulative tokens are introduced that weight transmissions according to channel availability and network congestion. Simulations show that dynamic allocation in virtual networks improves transmission performance, contributing to sustainability and cost reduction in cellular networks. This study highlights the importance of inefficient resource management by cognitive mobile virtual network and cognitive radio virtual network operators (C-MVNOs), laying a solid foundation for future applications in intelligent networks. This work is motivated by the increasing demand for efficient and scalable data transmission in smart metering systems. The novelty lies in integrating cumulative tokens and a Markovian-based bipartite graph matching algorithm, which jointly optimize channel allocation and transmission reliability under heterogeneous wireless conditions.

## 1. Introduction

The advanced metering infrastructure (AMI) supports the operation of a Smart Grid (SG). It includes smart meters (SM), communication networks for transporting data collected by the intelligent metering system to the utilities, a data management system, and, in some cases, a human-machine interface that allows users to monitor their energy consumption [1].

The communication infrastructure can employ different media, protocols, and technologies for SM monitoring, in addition to allowing remote management of subscribers’ service connection and disconnection. Today, smart metering devices play a key role in optimizing energy management systems, as they collect accurate information and transmit it efficiently over communication networks. Integrating communication functions in these devices has significantly improved energy monitoring, facilitating more efficient and reliable management of energy resources and reducing operating costs [2,3].

In this sense, wireless communication infrastructure for wireless sensor networks (WSN) has proven viable, as it reduces the costs associated with deploying physical infrastructure. In this sense, wireless SMs represent an application case of WSNs and, therefore, the Internet of Things (IoT) paradigm, where each SM is a sensor that collects consumption data from smart grid subscribers. Power consumption data can be monitored remotely through a WSN and its corresponding gateway [4,5].

The interest in connecting metering devices to the Internet has driven wireless cellular networks, which have become relevant in providing data access for users and smart metering systems [4]. These networks provide the primary communication infrastructure for AMI, enabling data transmission between smart meters and central data centers. However, MNO infrastructure leasing can often be costly, and in solutions where the number of metering devices is high, it can exponentially increase traffic demand [6]. For a small number of users, this may not be noticeable.

On the other hand, in wireless networks, virtualization technologies represent an innovative alternative to optimize, in particular, the use of infrastructure by creating virtual versions instead of physical ones. It makes it possible to share or reuse the physical resources of service providers, which facilitates the coexistence of multiple virtual networks on a unified infrastructure [7,8]. As a result, more efficient resource management and reduced operating costs are achieved. In addition, virtual wireless networks (WVN), facilitated by virtual network providers (VNBs), enable the extraction, segmentation, and sharing of physical infrastructure and radio resources. It makes them a suitable solution to meet the diverse requirements of future wireless networks [9,10].

**Figure 1:**
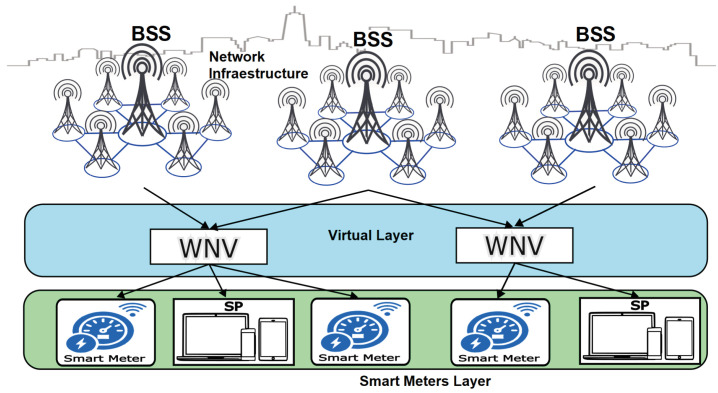
Allocation of virtualized resources in WNV [12].

Through WNV, a wireless sensor network of SMs can communicate efficiently with a set of base stations (BSS). This technology abstracts physical resources (spectrum and base stations) and presents them as customized virtual networks for different services, such as internet access for each sensor. This technology abstracts physical resources (spectrum and base stations) and presents them as customized virtual networks for different services, such as internet access for each sensor. Therefore, when a sensor transmits data, it does not send it directly to a specific base station but through the virtualized layer. This virtualized layer dynamically decides which base stations from the BSS set should receive and handle the traffic based on network load, channel quality, or service priority. Thanks to WNV, sensors can connect more flexibly and efficiently. The network assigns appropriate resources in real-time, allowing the system to scale the number of devices without overloading the physical infrastructure. Furthermore, if the sensor moves or conditions change, the network can seamlessly redirect it to another base station without interruptions [11]. Figure 1 presents a diagram illustrating a WNV scheme for managing network resources.

**Figure 2:**
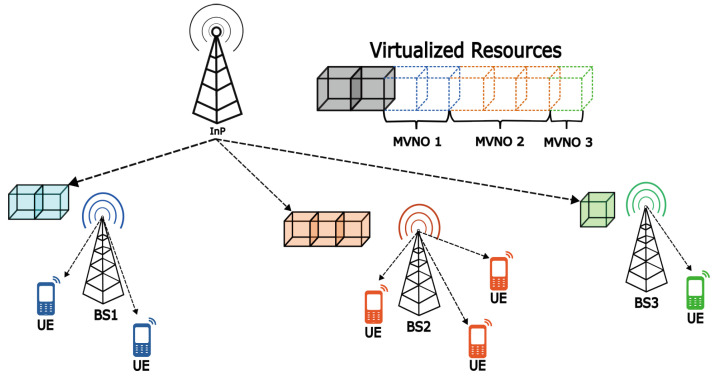
Virtualized network architecture.

The virtualization of a network offers an efficient alternative to reducing communication infrastructure costs in smart metering, which has become a key communication channel in this area. It highlights the need to efficiently manage channel allocation for these devices through wireless connectivity [11,13,14]. Figure 2 presents a diagram illustrating a wireless network virtualization (WNV) scheme for managing multiple MVNOs’ network resources.

Primary spectrum leasing is often mentioned when discussing mobile operators, while mobile virtual operators are associated with secondary spectrum leasing [12]. Spectrum resources are experiencing increasing scarcity due to the significant increase in mobile network users and wireless communication services.

Although licenses are allocated for most spectrum bands, these are often used inefficiently, even in densely populated urban areas, as mobile users do not always require radio resources [15,16]. Various methods of dynamic spectrum access have been proposed to address this issue and optimize the utilization of spectrum resources [17,18,19,20]. Cognitive radio and wireless network virtualization stand out.

Cognitive radio technology enables network operators to address spectrum constraints and inefficient spectrum usage by allowing unlicensed users to use available spectrum slots in licensed bands without affecting primary users. Competitive spectrum sharing among multiple secondary users in cognitive radio networks can also improve the utilization of limited radio spectrum [16].

The cognitive virtual mobile operator (C-MVNO) is an evolution of the MVNO concept that uses cognitive radio (CR) technology to improve channel utilization, reducing the costs associated with leasing primary spectrum from primary operators [16,21]. Unlike traditional MVNOs, which were required to lease all spectrum from the primary operator, C-MVNOs can allocate less spectrum by taking advantage of “white holes” in the primary licensed spectrum band [14]. This strategy allows them to use spectrum more efficiently and reduces dependence on spectrum assigned by the primary operator. Dynamic and opportunistic allocation of secondary network channels through a C-MVNO minimizes leasing costs by allowing flexible and opportunistic use of channels based on the wireless traffic demands of secondary unlicensed smart metering users [22,23]. However, how to jointly optimize cooperative channel sensing and spectrum access processes remains an open question, especially in the time-varying radio environment, although numerous distributed algorithms have been proposed in the literature [12,24].

This paper proposes a modeling approach to address the wireless channel resource optimization problem in the context of timely channel allocation in cellular wireless networks, specifically focusing on the SM reading.

Studies propose cascading machine learning algorithms that improve energy efficiency and dynamic channel allocation through intelligent predictions, providing a scalable and adaptable alternative for changing environments [25]. On the other hand, it focuses on multi-radio and multi-hop networks, proposing a joint strategy for channel allocation, power control, and route selection, which optimizes spectral efficiency and network capacity [26]. In addition, an adaptive scheme is introduced that combines channel allocation and routing, maximizing network performance through network coding and mechanisms for responding to spectrum variability [27]. In this context, a model for the joint allocation of subchannels, rates, and power for OFDM environments in cognitive networks is proposed. It considers both total power restrictions and those derived from distributed detection, which improves the coexistence between primary and secondary users. This work complements the proposal of the present article by offering novel and robust solutions for efficiently managing resources in dynamic and heterogeneous networks [28].

## 2. Related Works

Existing literature highlights the importance of optimizing communication infrastructure in advanced metering systems. Recent studies have explored techniques such as wireless network virtualization [7,8], the use of C-MVNO [12], and channel allocation algorithms based on graph theory [11]. These strategies have demonstrated advances in resource optimization, although challenges remain concerning channel detection and planning, as well as spectrum access in heterogeneous wireless environments. This work addresses these limitations by integrating cumulative tokens and a bipartite graph matching algorithm, optimized using the Hungarian algorithm, to enhance dynamic channel allocation and ensure transmission reliability in intelligent metering systems.

Smart meters are essential to energy management systems in SG as they enable real-time monitoring and efficient resource management. By incorporating communication functions, SMs improve reliability, optimize operational efficiency, and reduce costs, becoming a fundamental component of SG infrastructure [29,30].

Cellular networks provide a scalable solution for smart metering systems by leveraging their extensive coverage and existing infrastructure. This approach reduces implementation costs, enhances operational scalability, and supports large-scale deployments of AMI [31]. Mobile Virtual Network Operators (MVNOs), which establish agreements with primary operators, optimize smart meter communication through opportunistic wireless channel allocation and virtualization [9,32]. The use of channel allocation methods enables efficient utilization of available resources.

In this context, Cognitive Mobile Virtual Network Operators (C-MVNOs) expand the concept of MVNOs by incorporating cognitive radio technology, allowing for efficient spectrum utilization through the dynamic allocation of “white spaces” in licensed bands. This reduces leasing costs, improves spectrum management, and enhances transmission efficiency [8,9,14]. However, challenges such as random channel access and cooperative spectrum sensing highlight the need for more advanced algorithms to ensure sustainable resource allocation [22,33].

Device-to-device communication (D2D) is another interesting context in wireless communications systems that can contribute to the operation of smart metering systems [34,35]. D2D can significantly reduce latency and energy consumption by enabling direct communication between smart meters. In addition, it facilitates real-time energy monitoring and improves network efficiency by offloading traffic from the cellular infrastructure. In other words, D2D improves scalability and spectral efficiency, which would reduce costs for deploying a large-scale network of AMI [36]. However, implementing D2D in smart metering presents challenges such as interference between D2D and cellular users, the limited number of transmission channels, and the absence of accurate channel state information (CSI) [36].

In addition, efficiently managing channel allocation in dense environments remains a computationally complex task. Addressing these challenges requires advanced resource allocation algorithms, robust interference management, and scalable solutions like blockchain-based systems to ensure secure and efficient operations. Integrating D2D communication into C-MVNO frameworks underscores the potential for significant improvements in cost reduction, reliability, and sustainability but highlights the need for innovative strategies to overcome inherent limitations [37].

On the other hand, the use of the Non-Orthogonal Multiple Access (NOMA) technique in wireless communications systems allows the increase in the number of devices on a large scale, and its application in the IoT paradigm is shown as a high-impact solution to massify the connection of devices [38]. This is because NOMA allows multiple users to connect using the same channel by assigning different powers to each user on each channel [39]. However, although some proposals have been made to leverage NOMA in mass access networks, the communication system is highly complex in terms of processing and inter-cell interference. It is more sensitive to errors in channel estimation. This limits the number of users that can use a channel simultaneously. Therefore, using NOMA increases the need for proper planning of channel allocation for smart metering [40].

Table 1 summarizes the fields investigated in recent articles related to the topics discussed in this article. It is followed by a list of related reference works organized to provide a coherent and detailed overview of previous research in this field.

## 3. Problem Formulation and Methodology

**Figure 3:**
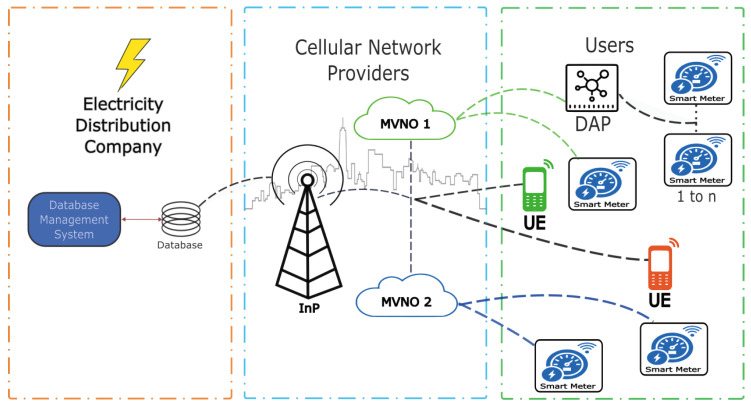
AMI architecture.

This section presents a model of a wireless cellular network system operated by a mobile virtual network operator (MVNO). The model simulates the data transmission from a specific number of smart meters (M) and the time (T) allocated for the transmission of read and write data from these devices. In addition, simultaneous access to voice and data services for cell phone users through a network of cells is included. Each cell is equipped with a carefully selected set of channels to avoid interference with adjacent cells. The smart meters are randomly distributed within the cell coverage area, and the channel assignment process follows a Markovian behavior. Figure 3 shows the AMI architecture of the proposed system.

In this scenario, each cell contains a set of smart meters that periodically require an uplink channel to transmit data. A mobile virtual network MVNO, which may be an independent entity such as an electric utility, is responsible for providing service to these smart meters. Then, to simplify the analysis, this paper focuses on a single-cell model that covers a number *M* of smart meters and has *N* channels available for transmission.

The analysis presented in this work proposes that the primary operator divides its available channels into three groups to ensure efficient resource allocation. The first group, consisting of $\left(N_{r}\right)$ fixed channels, is exclusively reserved for the operation of MNO users. The second group, comprising $\left(N_{f}\right)$ reserved channels, is dedicated to operating SMs. Finally, the third group includes the random channels $\left(N_{ra}\right)$, which can be used by both mobile operator users and smart counters through the MVNO, giving priority to the former. The allocation of these random channels depends on their availability. If the reserved channels are insufficient to meet AMI’s demand and there are free random channels, these will be allocated for use by smart meters. This division and prioritization scheme aims to maximize spectral efficiency while ensuring systems operate reliably and adaptively under varying load conditions. In addition, exclusive channels are established for the MNO’s general users to guarantee Quality of Service (QoS). For smart metering, channels are defined to be used through the MVNO to isolate massive device traffic and ensure the reliable transmission of AMI data. Finally, a group of random channels is reserved that can be used by MNO users or the MVNO based on demand and availability, with priority given to MNO users. The total available channels is $N=N_{r}+N_{f}+N_{ra}$.

**Figure 4:**
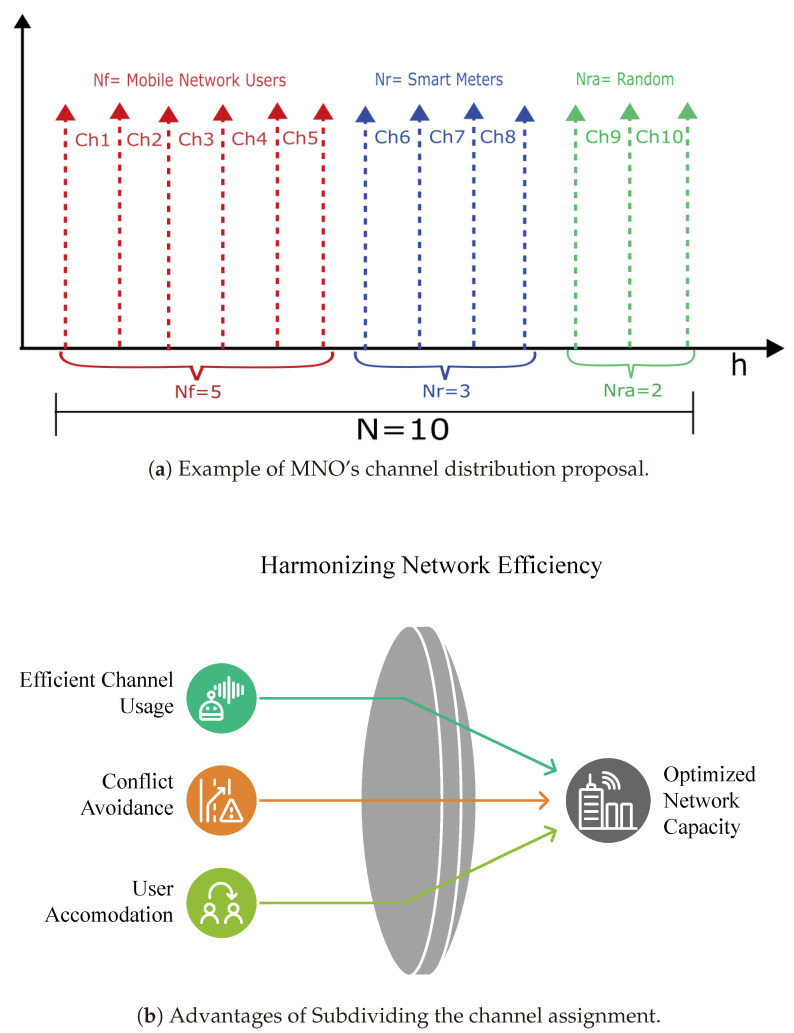
Channel distribution in a communication network for smart metering.

Figure 4 presents an illustrative example of allocating communication channels with *N* = 10 in an AMI. In this case, the channels are organized into three main categories:

- Mobile Network Users ($N_{f}=5$, in red): Represent the mobile network users to transmit data. In this example, the assigned channels correspond to Ch1 to Ch5.
- Smart Meters ($N_{r}=3$, in blue): Correspond to electrical metering devices that send information to the central system. The channels assigned to this category are Ch6 to Ch8.
- Random channels ($N_{r}a$ = 2, in green): These are assigned to occasional unplanned connections or users. The corresponding channels are Ch9 and Ch10.

In Figure 4a, the abscissa axis corresponds to channel allocation, red identifies the MNO’s fixed channels for its users, blue identifies the channels reserved for MVNO use, and green identifies the random channels that can be used by the MNO or the MVNO depending on availability. This example reflects how network resources are distributed to optimize network capacity, ensuring connectivity for users and devices with different priority levels. Figure 4b summarizes the advantages of segmenting MNO channels to use smart metering through the MVNO.

A time slot is the interval required for the smart meter to transmit consumption information. In a given time slot, $n_{c}$ channels can be active and used by mobile network users. These users are limited in each cell by the sum of reserved channels and random channels, i.e., $n_{c}\leq N_{r}+N_{ra}$. Additionally, $n_{a}$ smart meters can be scheduled to be active during that period; however, not all active meters will necessarily transmit information. Only $n_{a}^{*}$ smart meters will effectively transmit, where $n_{a}^{*}$ is the minimum between the number of scheduled active meters $n_{a}$, the number of channels available for SMs, calculated as $N-n_{c}$ (the total number of channels minus those used by mobile network users), and the sum of fixed and random channels $N_{f}+N_{ra}$. Therefore, the equation for $n_{a}^{*}$ is given by $n_{a}^{*}=\min\left\{n_{a},N-n_{c},N_{f}+N_{ra}\right\}$, which ensures that channels are used efficiently and that there are no transmission conflicts.

The transmission of smart meters on reserved channels is always assured, whereas, on random channels, it depends on availability, i.e., the transmission may or may not take place and is conditioned to a certain probability. According to the real-time situation, the model attributes to the primary cellular operator the management of when and on which channel smart meter transmissions will occur. The transmission schedule is defined by an algorithm called SchAlg, which assigns each smart meter a time slot and channel for transmission. The periods are organized into *T* frames, each with multiple time slots. The scheduling is recalculated for each time interval and can be represented by a binary variable $x_{h,t,k}=1$ if the *k*-th smart meter uses the time interval *t* within channel *h* to carry out the transmission.

Therefore, its scheduling must comply with certain constraints defined in the Equation (1).

(1) $$\sum_{h=1}^{N}\sum_{k=1}^{M}x_{h,t,k}\leq 1;\;\forall t\in \left\{1,2,\ldots ,T\right\}$$

So that only one smart meter is scheduled on the same channel and time interval. The constraint $\sum_{h=1}^{N}\sum_{t=1}^{T}x_{h,t,k}\leq 1;\;\forall k\in \left\{1,2,\ldots ,M\right\}$ indicates that a smart meter is scheduled to transmit in only one-time interval and one channel. Each smart meter has a preferred time interval for transmission, denoted as $t_{k}$, corresponding to a specific moment within the transmission frame.

However, the smart meter can perform transmissions in a different interval within a transmission frame $\left[t_{k}-W/2,t_{k}+W/2\right]$, where *W* represents the window length. This flexibility helps to avoid network congestion. The variable is established as $z_{h,t,k}\leq x_{h,t,k}$, where $z_{h,t,k}\in \left\{0,1\right\}$, indicating whether the smart meter has effectively transmitted the information for the scheduled combination of channel and time interval. This variable represents an additional condition or a particular state associated with the successful transmission of the *k*-th smart meter’s information in channel *h* and time interval *t*.

The scenario also considers a set of transmission frames F={1,2,…} and the variable $z_{f,k}=\sum_{h=1}^{N}\sum_{t=1}^{T_{f}}z_{h,t,k}$, which acts as an indicator, taking the value of one when smart meter *k* effectively transmits in frame *f*. It is important to note that $t_{f}$ is the time interval associated with frame *f*. The average user transmission throughput is defined as $TH_{k}=\lim_{F\to \infty }\frac{1}{F}\sum_{f=1}^{F}z_{f,k}$. Likewise, the total throughput of the smart meter cell is defined as $TH=\lim_{F\to \infty }\frac{1}{F}\sum_{f=1}^{F}\sum_{k=1}^{M}z_{f,k}$.

Ideally, these values should approach a limit of $TH_{k}\to 1:M$; these quantities measure system performance. In this context, the selected values impact the factors of the global performance metrics. The objective is to identify these effects and establish the relationship between the performance metrics, as defined, and the system variables, which include *N*, $N_{f}$, $N_{r}$, $N_{ra}$, *M*, *h*, and *t*.

This paper’s proposal is based on a bipartite minimum weight problem, in which the various smart meters represent the nodes that have to be allocated to a specific combination of time slots and channels, which we will call a resource unit. This allocation process can be represented as a bipartite network structure, with the smart meters in one set and the resource blocks grouped in another. The assigned weights and constraints between nodes are then established. A constraint is set between the $k_{th}$ smart meter and a resource block (h,t) if *t* is within the transmission window of the smart meter. The weight, represented by $W_{t,h}^{k}$, is set according to the criteria below:

- The temporal distance weight, denoted as $w_{t,k}$, depends on the distance between the time interval *t* and the preferred time interval $t_{k}$. This weight is minimal for the value of $t_{k}$ and increases as the distance from $t_{k}$ increases. A shorter distance implies a higher likelihood of successful transmission, resulting in a lower weight value.
- The channel weight, denoted as $w_{h,k}$, depends on the chosen channel. If *h* belongs to the set of fixed channels, its value is lower since the probability of successful transmission on this channel is higher. Similarly, if *h* belongs to the set of random channels, its value is higher because the transmission probability on these channels is lower than on fixed channels.
- The fairness weight, denoted as $w_{k}$, aims to promote an adequate allocation of resources to smart meters. $w_{k}$ is a value based on **tokens**. If a smart meter has not transmitted over several frames, it accumulates tokens corresponding to each. When the smart meter transmits successfully, no additional tokens are added. The value $w_{k}$ is the inverse of the number of accumulated tokens; thus, the more tokens accumulated, the lower the fairness weight favors resource allocation.

The product of the three weights, $w_{t,k}$, $w_{h,k}$, and $w_{k}$, calculates the final weight value. This result is lower for smart meters with lower throughput, making resource allocation more favorable. It will also be lower in time intervals closer to the preferred transmission interval and even lower on reserved channels.

Finally, the problem is solved as a minimum-weight bipartite matching problem using the Hungarian algorithm, which defines the final scheduling. This scheduling is implemented, and the parameters $z_{h,t}^{f,k}$ are determined as input for the next frame. This process is applied iteratively in subsequent frames. The algorithm is presented in Algorithm 1.

Additionally, evaluating the transmission loss rate in a smart metering network is considered, where each device attempts to communicate data during its preferred time interval $t_{k}$. This interval follows a probabilistic model, in which each transmission attempt may be successful or failed depending on network congestion. If an SM fails to transmit successfully in its assigned interval, a token is accumulated, which affects its priority in future attempts within the transmission window *T*.

This analysis covers several scenarios by varying the number of meters *M*, the window length *T*, the number of random access channels $N_{ra}$, and the number of reserved channels $N_{r}$. In addition, the impact of smart meter traffic on transmission probability is assessed, analyzing whether network congestion causes an increase or decrease in token accumulation. Specifically, it is studied whether more transmissions are lost under higher traffic conditions, resulting in more accumulated tokens. The probability of successful transmission also varies depending on how congested the primary operator is, directly influencing network performance.

The simulation time will be limited, assuming this time corresponds to the period a packet can remain in a memory buffer waiting to be transmitted. Therefore, if it is determined that an SM has accumulated *T* tokens, this is equivalent to having lost *T* transmission packets.

The model incorporates the token accumulation criterion to analyze the loss rate and the average number of tokens accumulated per smart meter. Each SM *k* starts with no accumulated tokens at the beginning of the simulation, i.e., $T_{k}\left(0\right)=0$. If an SM fails to transmit successfully in its preferred interval $t_{k}$, one token is added to its count for the next attempt, represented by the equation $T_{k}\left(n+1\right)=T_{k}\left(n\right)+1$, where *n* denotes the number of failed attempts.

The transmission weight $W(T_{k})$ is adjusted based on the number of accumulated tokens $T_{k}$ and is expressed by the formula $W\left(T_{k}\right)=\frac{1}{1+T_{k}}$. This relationship implies that as an SM accumulates more tokens, its transmission weight decreases, increasing its probability of transmitting in the next attempt. The transmission weight is also affected by the congestion level of the primary operator, which can cause this weight to increase or decrease depending on the network situation.

In summary, the fundamental aspects of the model are as follows:

- Initial Token: Each SM *k* starts with no accumulated tokens at the beginning of the simulation, i.e., $T_{k}\left(0\right)=0$.
- Token Increment: If an SM fails to successfully transmit during its preferred interval $t_{k}$, one token is added to its count for the next attempt, expressed as $T_{k}\left(n+1\right)=T_{k}\left(n\right)+1$, where *n* represents the number of failed attempts.
- Transmission Weight: The transmission weight $W(T_{k})$ is adjusted according to the number of accumulated tokens $T_{k}$ and is given by the formula $W\left(T_{k}\right)=\frac{1}{1+T_{k}}$. This relationship reduces the transmission weight for SMs with more tokens, thereby increasing transmission probability for those with higher accumulation in the next attempt. The probability of successful transmission is also influenced by the congestion level of the primary operator, which may cause this weight to increase or decrease depending on the network conditions.

**Figure 5:**
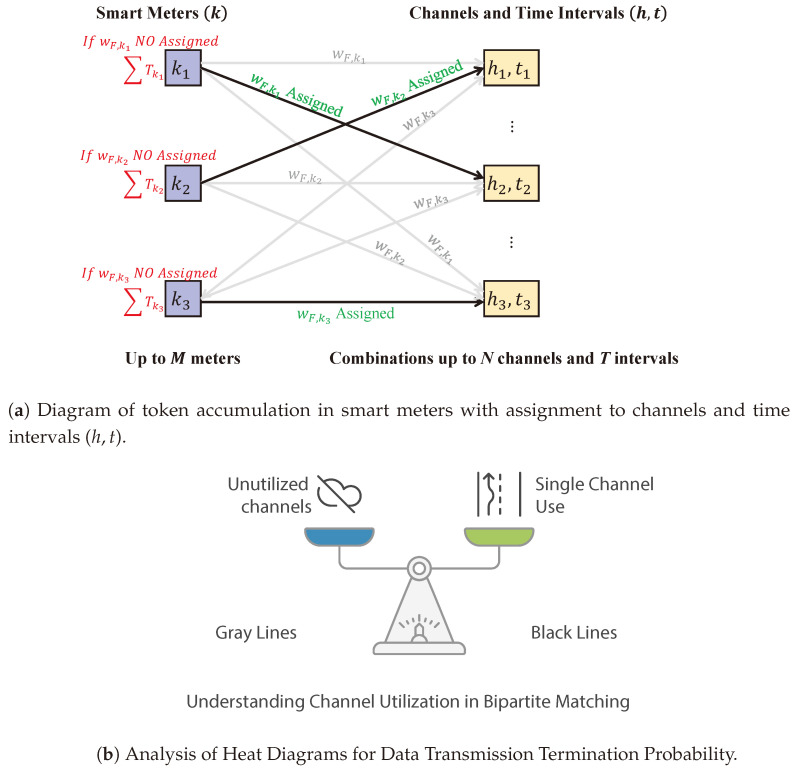
Channel distribution in a communication network for smart metering.

Figure 5a shows a simplified model schematic illustrating the minimum-weight bipartite matching problem optimized by the Hungarian algorithm, presenting the most optimal resource allocation in each iteration. Resource weights from the optimization process are highlighted in green (*w*_*F,k*_ Assigned), and assigned edges are depicted in black. Gray lines indicate that an SM did not establish a connection using the corresponding channel marked in gray. On the other hand, each block on the left side of the diagram has only one black line emerging, signifying that each user can utilize only one channel for connection.

Accordingly, Figure 5b represents choosing one channel or another according to the weight calculated through the Hungarian algorithm.

Since $t_{k}$ is modeled as an event with a probability of success, the system exhibits characteristics of a Markovian process. In each time interval *T*, the transmission probability for each SM depends solely on its current state $T_{k}$, without considering previous states. Thus, to analyze the steady-state behavior of the system, the average transmission loss rate and the cumulative token behavior are computed over several *s* = 100 simulations. Then, the system’s state is considered to calculate the average number of accumulated tokens over 100 simulations. Let $T_{k}^{\left(j\right)}$ be the number of tokens accumulated by meter *k* in simulation *j*, where j=1,2,…,100.

Thus, the system’s state in each simulation is considered to calculate the average number of accumulated tokens over 100 simulations. Let $T_{k}^{\left(j\right)}$ represent the number of tokens accumulated by meter *k* in simulation *j*, where j=1,2,…,100. The average number of accumulated tokens for SM *k* over 100 simulations is denoted as ${\bar{T}}_{k}$, and is calculated using the formula ${\bar{T}}_{k}=\frac{1}{100}\sum_{j=1}^{100}T_{k}^{\left(j\right)}$, where ${\bar{T}}_{k}$ represents the average number of tokens accumulated by meter *k* across the 100 simulations. Furthermore, if the total average number of tokens accumulated in the network for all SMs k=1,2,…,K is desired, it is given by the expression ${\bar{T}}_{\mathrm{total}}=\frac{1}{K}\sum_{k=1}^{K}{\bar{T}}_{k}=\frac{1}{100\cdot K}\sum_{k=1}^{K}\sum_{j=1}^{100}T_{k}^{\left(j\right)}$.

Table 2 describes the variables used in the mathematical model and the algorithm.

The procedure of the model is presented in Algorithm 1.
**Algorithm 1** Transmission allocation algorithm with token accumulation averaged over 100 simulations.**Require:** *M*, *N*, *T*, *W*—Input variables**Ensure:** TH—Output variable1:  **Step 1:** Calculate $w_{t,h,k}$ for all k∈{1,…,M}, t∈{1,…,T}, h∈{1,…,N}2:           $w_{t,h,k}=f\left(k,t,h\right)$3:  **Step 2:** Set $x_{h,t,f,k}$ from solution of minimum weight bipartite matching problem4:           $x_{h,t,f,k}=\arg\min_{x}\sum w_{t,h,k}$5:  **Step 3:** Solve cellular channel occupancy during the frame6:           Channel occupancy = g(h,t)7:  **Step 4:** Set $z_{h,t,f,k}$ from channel occupancy8:           $z_{h,t,f,k}=\left\{\begin{array}{cc} 1 & \mathrm{if}\;\mathrm{channel}\;h\;\mathrm{is}\;\mathrm{available}\;\mathrm{for}\;k\\ 0 & \mathrm{otherwise} \end{array}\right.$9:  **for** j=1 **to** 100 **do**10:      **for all** k∈{1,…,M} **do**11:            **Step 5:** $z_{t,f,k}=\sum_{h=1}^{N}z_{h,t,f,k}$12:            **if** $z_{t,f,k}==0$ **then**13:                 **Step 7:** ${\mathrm{Token}}_{k}\leftarrow {\mathrm{Token}}_{k}+1$14:            **end if**15:      **end for**16:      **Step 8:** Weight Update17:              For each k∈1,...,M:18:                      Calculate $wk=1/(1+T_{k})$19:                      Calculate wt,k based on temporal distance20:                      Calculate wh,k based on selected channel21:                      $w_{F,k}=w_{k}*w_{t,k}*w_{h,k}$22:      **Step 9:** Record total tokens for each *k* in simulation *j*23:**end for**24:**Step 10:** Compute the average tokens accumulated for each *k* over 100 simulations25:         ${\bar{T}}_{k}=\frac{1}{100}\sum_{j=1}^{100}T_{k}^{\left(j\right)}$

## 4. Analysis of Results

The proposed simulation was implemented on a computer with a 2.6 GHz Intel Xeon processor, 64 GB of RAM, and a 500 GB SSD disk. Matlab R2023b software was used for the development. Regarding computational complexity, the Big O notation of the Hungarian algorithm used in the model is $O(n^{3})$.

Then, to evaluate the performance of the proposed model, 40 simulation scenarios have been obtained that vary according to three primary parameters: the probability of an inactive meter initiating a transmission ($p_{a}$), the likelihood of disconnection of an active meter ($p_{d}$) and the number of reserved channels ($N_{r}$). These combinations generate specific configurations that allow analysis of the impact of variations in network congestion and transmission efficiency.

Table 3 summarises the main characteristics of the simulated scenarios, grouped into two groups of 20 scenarios each. The first block, corresponding to Group 1 ($p_{a}=0.001$), represents a low congestion environment, while the second block, corresponding to Group 2 ($p_{a}=0.01$), models a scenario with higher network traffic. Each scenario combines different values of $p_{d}$ and $N_{r}$ to cover various configurations in smart meter data transmission systems.

**Figure 6:**
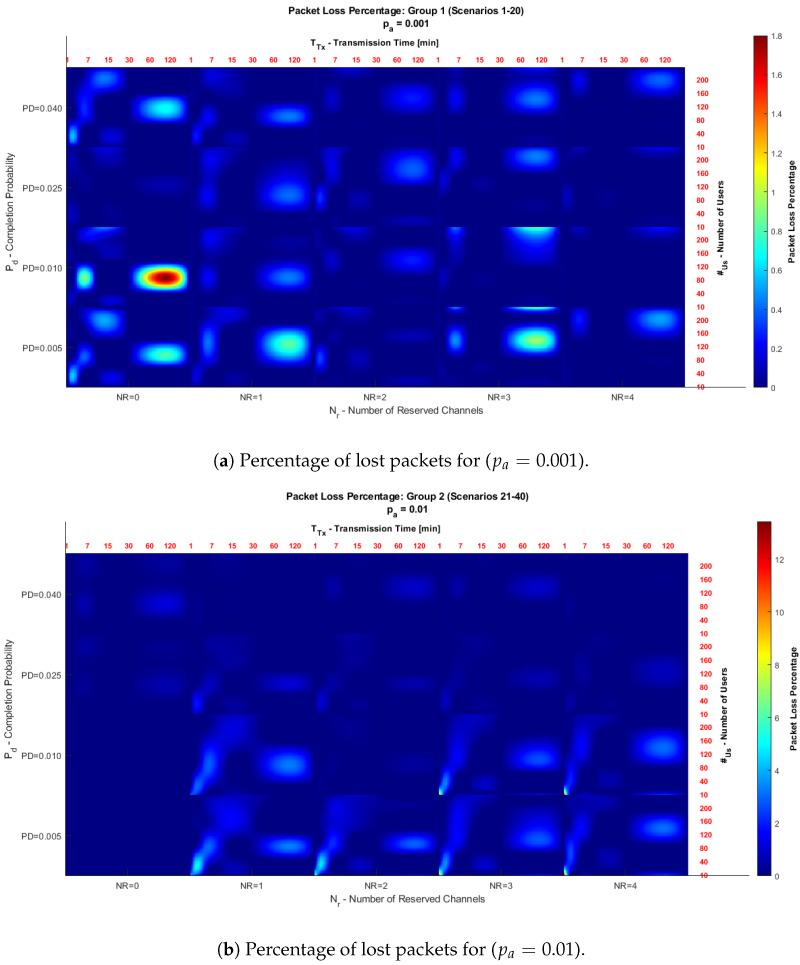
Comparison of the percentage of lost packets for $p_{a}=0.001$ and $p_{a}=0.01$.

**Figure 7:**
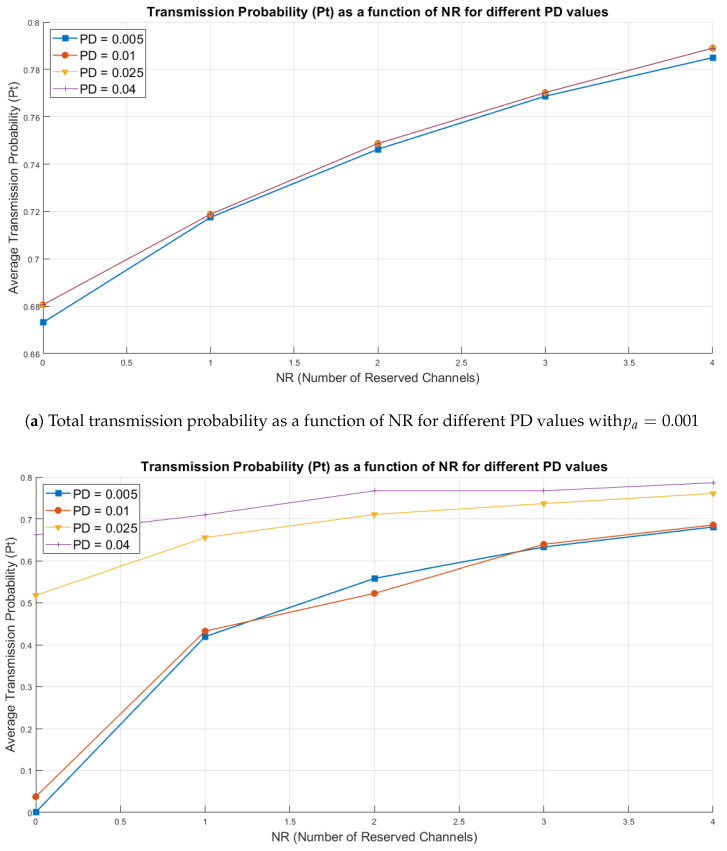
Comparison of transmission probability between $p_{a}=0.001$ and $p_{a}=0.01$.

**Figure 8:**
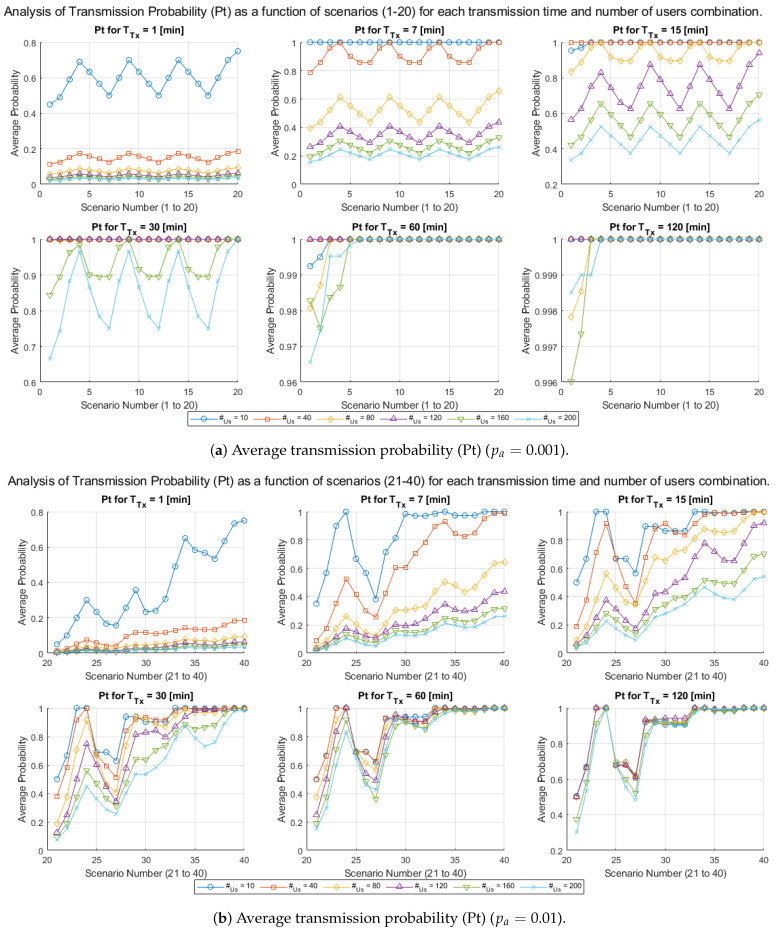
Average transmission probability (Pt) as a function of the number of scenarios processed, under two congestion levels $p_{a}=0.001$ and $p_{a}=0.01$.

In the analysis performed, heat plots (Figure 6) are presented that illustrate the relationship between reserved channels ($N_{r}$) and the probability of disconnection ($p_{d}$) in terms of the percentage of packets lost. The sub-figures associated with each coordinate ($N_{r},p_{d}$) show, for each scenario, how the transmission time parameters (vT) and the number of smart meters (vM) interact. Additionally, line graphs (Figure 7 and Figure 8) are included to analyze the average transmission probability (Pt) as a function of $N_{r}$, vT and vM. These metrics allow trends to be identified and provide insight into the system’s behavior under different traffic conditions.

The scenario parameters obtained in the simulation are shown in the Table 3:

Figure 6 shows two heat diagrams illustrating the relationship between the reserved channels (Nr) and the data transmission termination probability (pd). Each coordinate, defined by the values of Nr and pd, is composed of sub-figures showing, for a specific scenario, the relationship between vT or transmission times ($T_{\mathrm{Tx}}$) on the x-axis, and vM, which corresponds to the number of subscribers (#Us), i.e., the number of smart meters connected to the network, on the y-axis.

The color scale in the diagrams indicates the percentage of packets lost: warmer shades reflect higher losses, while more fabulous shades represent lower losses.

It is important to note that the scale of the heat diagrams was adjusted to facilitate the interpretation of the results. In Figure 6a, the scale was adjusted to a range of 1 to 1.8, while in Figure 6b, a range of 1 to 14 was used. This change allows for better visualization and comparison of the behavior in each case.

Figure 6a shows that the areas with warm shades, such as red and yellow, correspond to parameters where the model has a higher percentage of lost packets. In contrast, areas in calm tones, such as blue and white, represent configurations with lower losses. The results indicate that as the number of reserved channels (Nr) increases, the system improves its stability, decreasing the percentage of losses.

The disconnection probability (pd) also impacts the results. As pd increases, the percentage of lost packets decreases, indicating that a higher probability of terminating transmissions allows channels to be released more quickly. It reduces congestion and favors the transmission of remaining packets. In this Figure, with pa=0.001, the probability of an idle meter initiating a transmission is low, resulting in less traffic on the network. Therefore, overall packet losses are lower compared to higher traffic situations. In addition, areas are identified at the origin where no packet losses are recorded in configurations with Nr=0 and a low call termination probability (pd). In these conditions, the transmission probability tends to zero, which implies that no transmission is made and, therefore, no packet loss percentage is recorded.

Figure 6b shows similar behavior to Figure 6a, but with a higher intensity in the percentage of lost packets. This increase is because, as pa=0.01 increases, the probability of an inactive meter initiating a transmission increases, which causes an increase in network traffic and, consequently, a higher percentage of lost packets.

Despite the increase in congestion, the general trends remain the same. As Nr increases, losses decrease, highlighting the importance of reserving channels to efficiently manage traffic on the smart meter transmission network. Furthermore, it is observed that high values of pd contribute to reducing losses by allowing to terminate transmissions, freeing resources for new requests.

According to the Figures obtained, it can be evidenced that:

- Effect of $p_{a}$: In Figure 6b ($p_{a}=0.01$), the percentage of lost packets is higher than in Group 1 ($p_{a}=0.001$). This is due to the increased traffic caused by a higher transmission probability from inactive meters.
- Effect of $N_{r}$: In both cases, having reserved channels ($N_{r}>0$) is essential to reduce losses, especially in highly congested scenarios.
- Effect of $p_{d}$: A higher disconnection probability ($p_{d}$) reduces the percentage of lost packets, as it frees up resources more quickly, enabling more efficient handling of transmissions.

Figure 7 presents two line Figures showing the relationship between the number of reserved channels (Nr) and the average data transmission probability (Pt) for different values of transmission termination probability (pd). Each line represents a specific configuration of pd, allowing us to analyze how Pt varies as a function of Nr.

The x-axis shows the number of reserved channels (Nr), while the y-axis represents the average transmission probability (Pt). The colors and line styles differentiate the different termination probabilities (pd), allowing a visualization of behavioral trends. These figures will enable us to identify how the system responds to changes in channel reservation and disconnection probability under different congestion levels.

In Figure 7a, it can be observed that the transmission probability ($P_{t}$) improves significantly as the number of reserved channels ($N_{r}$) increases. With $p_{a}=0.001$, the probability that an inactive meter initiates a transmission is low, resulting in a less congested network environment. This scenario allows the system to operate with greater stability and efficiency in resource allocation, which is reflected in higher values of $P_{t}$. Figure 7a shows that for cases with PD = 0.01 and beyond, the average Transmission Probability remains constant, causing the curves for PD = 0.01 (red), PD = 0.025 (yellow), and PD = 0.04 (purple) to overlap each other.

The disconnection probability ($p_{d}$) also has a considerable impact. As $p_{d}$ increases, the system releases resources more quickly by terminating active transmissions, enhancing overall network efficiency. This effect is more evident when $N_{r}>0$, demonstrating that channel reservation helps maintain high levels of $P_{t}$, even in scenarios with increased traffic.

On the other hand, configurations without reserved channels ($N_{r}=0$) show significantly lower $P_{t}$ values, highlighting that the absence of reservation negatively affects the system’s ability to manage concurrent transmissions, even in a network environment with lower traffic.

In Figure 7b, a behavior similar to that observed in Figure 7a is noted, but with a lower transmission probability ($P_{t}$) due to an increase in $p_{a}=0.01$. This increase in $p_{a}$ causes more inactive meters to start transmitting, leading to higher network congestion and making efficient resource management more difficult.

Despite the increase in traffic, the trend of improvement in $P_{t}$ as $N_{r}$ increases still persists. It again highlights the importance of having reserved channels to mitigate the effects of congestion. Moreover, higher values of $p_{d}$ significantly reduce congestion by allowing active transmissions to end more quickly, freeing up resources to handle new requests.

However, in scenarios without reserved channels ($N_{r}=0$), $P_{t}$ is noticeably low, especially with $p_{a}=0.01$, reinforcing the need for proper resource management in high-traffic scenarios.

### Comparison of Figures

The evaluation performed between Figure 7a,b shows that:

- Effect of $p_{a}$: In Figure 7b ($p_{a}=0.01$), the average transmission probability ($P_{t}$) is lower compared to Figure 7a ($p_{a}=0.001$), due to increased traffic caused by a higher transmission probability from inactive meters.
- Effect of $N_{r}$: In both figures, increasing $N_{r}$ substantially improves the $P_{t}$ values. It demonstrates that allocating reserved channels is crucial to maintaining high system performance, particularly under high congestion conditions.
- Effect of $p_{d}$: A higher disconnection probability ($p_{d}$) promotes resource release, increasing system efficiency and enabling more effective data transmission. This effect is more evident in Figure 7b, where the high traffic load amplifies the importance of $p_{d}$ in managing congestion.

Figure 7a,b highlight the relevance of efficient management of reserved channels ($N_{r}$) and disconnection probability ($p_{d}$) to optimize the average transmission probability ($P_{t}$). While $p_{a}=0.001$ represents a more stable environment with lower traffic, $p_{a}=0.01$ significantly increases congestion, requiring greater system capacity to maintain high $P_{t}$ levels.

Figure 8 presents two charts containing a set of lines illustrating the relationship between the number of processed scenarios and the average data transmission probability ($P_{t}$). The scenarios are divided into two groups of 20: Group 1 ($p_{a}=0.001$), which includes scenarios 1 to 20 and represents low-congestion conditions, and Group 2 ($p_{a}=0.01$), which provides for scenarios 21 to 40 and represents high-congestion conditions in the cellular network. These charts allow the analysis of $P_{t}$ for different combinations of transmission time (vT) and number of smart meters (vM).

Each line represents a specific configuration of vM, allowing analysis of how $P_{t}$ varies depending on the values of vT and the number of processed files.

The *x*-axis shows the number of analyzed scenarios, while the *y*-axis represents the average transmission probability ($P_{t}$). Line colors and styles differentiate the vM values, allowing a straightforward visual analysis of the generated trends. These charts will enable us to identify how the model responds to changes in transmission parameters (vT) and smart meter density (vM) under different traffic scenarios, such as those defined by $p_{a}=0.001$ and $p_{a}=0.01$.

In Figure 8a, a low-congestion environment is observed due to the low transmission initiation probability ($p_{a}=0.001$). The results obtained show the following:

- General Trend: It can be observed that $P_{t}$ remains relatively stable for most vT values, especially when vM is high (vM=160 or vM=200). Lower values of vT (e.g., vT=1 or vT=7) exhibit slight fluctuations in $P_{t}$, but these are less significant compared to higher vT values. Additionally, it is observed that for low vT values, $P_{t}$ increases considerably when $N_{r}$ is high, leading to a “sawtooth” pattern, clearly visible in the subfigures with $T_{\mathrm{Tx}}=7$ and $T_{\mathrm{Tx}}=15$ minutes, respectively. This behavior is due to the rapid release and occupation of reserved channels, which causes periodic fluctuations in the transmission probability, particularly in scenarios with lower data traffic.
- Impact of vM: As vM increases, $P_{t}$ values decrease, highlighting a loss of transmission efficiency when the system handles a more significant number of active meters.
- System Stability: The average transmission probability ($P_{t}$) shows low variability across the different scenarios, indicating predictable and efficient behavior, particularly under low-congestion conditions.

Figure 8b represents a more congested scenario due to the increase in $p_{a}$ ($p_{a}=0.01$). It allows us to observe that:

- General Trend: $P_{t}$ exhibits greater fluctuations between the files, especially for low vT values (e.g., vT=1 and vT=7). These fluctuations reflect higher traffic levels in the system. On the other hand, for high vT values (e.g., vT=120 and vT=200), $P_{t}$ shows a clearer upward trend, highlighting the importance of longer transmission times to mitigate the effects of congestion.
- Impact of vM: As in Figure 8a, as vM increases, $P_{t}$ values decrease, indicating a loss of transmission efficiency when the system operates with a more significant number of active meters.
- Efficiency under Congestion: Although $P_{t}$ is generally lower in Figure 8b compared to Figure 8a, scenarios with high vT and vM values are able to approach higher efficiency levels.

The comparison between Figure 8a and Figure 8b shows that:

- Impact of $p_{a}$: In Group 1 ($p_{a}=0.001$), the system operates in a lower congestion environment, resulting in higher and more stable values of $P_{t}$. In contrast, in Group 2 ($p_{a}=0.01$), the additional traffic causes more significant fluctuations and a decrease in the average transmission probability ($P_{t}$).
- Effect of vT: In both figures, $P_{t}$ improves significantly with higher vT values, highlighting that longer transmission times allow for more efficient use of the channels.
- Effect of vM: High vM values negatively impact system performance, as $P_{t}$ decreases as vM increases, regardless of the group considered. In Group 2, where $p_{a}=0.01$, this effect is more pronounced due to increased traffic, intensifying congestion and reducing system efficiency. It underscores the importance of properly managing the number of active smart meters (vM) to minimize its impact on the average transmission probability.
- Variability Across Files: The variability of $P_{t}$ across the files is significantly higher in Group 2, highlighting the impact of the increased $p_{a}$ on system stability.

Figure 8a,b highlight the importance of optimizing the parameters vT and vM to ensure efficient system performance, particularly in high-congestion scenarios ($p_{a}=0.01$). While Group 1 exhibits more stable and predictable behavior, Group 2 introduces additional challenges that require specific conditions (e.g., vT>100 and vM≥160) to maintain adequate levels of average transmission probability ($P_{t}$).

These results demonstrate that carefully designing system parameters can mitigate the adverse effects of high traffic, optimize efficiency, and reduce performance fluctuations.

These observations provide a solid foundation for optimization decisions in cellular network resource management, especially in environments involving traffic from networks that support AMI over MVNOs. Possible optimization strategies include dynamic channel allocation, prioritization of critical transmissions based on their importance, or adjusting parameters such as $N_{r}$ and $p_{d}$ to mitigate congestion in high-traffic scenarios.

## 5. Conclusions and Discussion

This paper shows the application of an optimization model for resource allocation in cellular networks, considering different traffic and transmission conditions. It is identified that the number of reserved channels (Nr) has a considerable influence on system performance since its increase reduces the percentage of lost packets (paktPorcentual) and improves the average transmission probability (Pt). It demonstrates the importance of reserving sufficient resources to mitigate congestion in high-demand scenarios.

The disconnection probability (pd) directly impacts system efficiency since freeing up transmission channels more quickly allows new transmission requests to be accommodated. This effect is observed in both loss reduction and system stability.

Also, it is identified that the probability of new information sending pa directly impacts the system congestion. While Group 1 (pa=0.001) operates in a more stable and efficient environment, Group 2 (pa=0.01) shows a lower performance due to the increase in transmission traffic. Therefore, this has allowed modeling the case where the number of transmissions rises, for example, due to a possible connection failure and where the smart metering system sensors stored information that they could not send due to lack of connection; this network traffic rises with interest when the number of users increases.

The parameters of transmission time (vT) and the number of smart meters (vM) are determinants of system performance. High values of vT and vM require specific configurations to ensure adequate performance, especially in high-congestion scenarios.

The model demonstrates that considering an appropriate transmission time is critical for efficient system performance, as it directly impacts the transmission probability (Pt) and the network’s ability to meet the needs of the SM. This result highlights the importance of establishing optimal transmission times according to the required load profile, ensuring the desired resolution quality and efficient use of network resources.

The indicators show that proper design of system transmission parameters can mitigate the adverse effects of high traffic, optimizing efficiency and reducing performance fluctuations.

The results of this study underline the relevance of efficient resource management in cellular networks to maximize system performance. The variation of the probability of initiating a data transmission (pa=0.001 and pa=0.01) shows that, although the increase in the initial transmission probability increases the traffic in the network, its adverse effect can be compensated by an appropriate configuration of the system parameters, i.e., by obtaining a more significant number of channels reserved for AMI system communication.

The “sawtooth” behavior at low values of vT reveals a high dependency of the system on reserved channels. This is evidenced by the significant increase in the transmission probability as these resources increase and the sharp drop in the transmission probability as these resources decrease. This pattern attenuates with increasing transmission times, which allows the system to stabilize more quickly.

On the other hand, the results highlight the importance of high values of Nr and pd to maintain optimal performance, particularly in higher traffic scenarios. These parameters not only improve system stability but also allow for a more efficient use of available resources.

However, the results also reveal the inherent behavior of the system in configurations with high values of vM, where congestion and losses increase. It highlights the importance of implementing adaptive strategies that efficiently manage scenarios characterized by high user density.

Based on observing the system behavior in the different scenarios, reserved channels are essential to achieving optimal performance in smart meter reading through an MVNO. This strategy efficiently manages transmissions, reduces network congestion, and minimizes packet losses, leading to reliable system performance.

In practical terms, these results are relevant for evaluating the design of timely channel allocation in cellular networks, focusing on smart meter network applications. The proposed model provides a solid foundation for future research exploring advanced optimization strategies in dynamic and congested systems.

For future work, these results are relevant for evaluating the design and timely allocation of channels in cellular networks, focusing on applications such as AMI. Implementing adaptive strategies, such as dynamic resource allocation algorithms or machine learning, to predict congestion patterns could enhance system efficiency and flexibility. Furthermore, integrating emerging technologies, such as 5G networks, offers a promising way to optimize performance in high traffic variability and operational complexity scenarios. Moreover, it is relevant to analyze the impact of the proposed configurations on energy efficiency, as efficient use of resources improves the quality of service and minimizes the energy consumption of devices and infrastructure. In this context, it is recommended to evaluate the use of data aggregation points (DAPs) as an alternative to improve performance in suburban and rural solutions, allowing us to assess the integration of these scenarios and their impact on the cellular network. Finally, pilot tests should be implemented in real networks to validate the proposed model’s practical feasibility, especially in dynamic and congested environments. These tests will allow for fine-tuning system parameters, confirming its effectiveness and ensuring its applicability in real-world scenarios. These areas represent key lines for future research and the development of innovative solutions in cellular networks, particularly in critical applications such as smart metering.

On the other hand, complementary future work is identified as applying NOMA to define how much user capacity can be increased by having several users per channel simultaneously and to evaluate how many users per channel can be accepted in the context of smart metering for different user scales.

## Figures and Tables

**Table 1:** Summary of related works.

Author	Optimization	Scheduling	Cost	Matching	C-MVNO	D2D	NOMA
Abdulsalam, 2023 [2]			✓				
Nomikos, 2022 [3]	✓		✓	✓			
Adebayo, 2022 [11]	✓		✓	✓			
Guan, 2022 [13]	✓			✓			
Inga, 2022 [14]	✓		✓	✓	✓		
Ren, 2022 [7]	✓			✓			
Zhang, 2022 [8]	✓		✓	✓			
Li, 2022 [41]	✓	✓	✓	✓			
Liu, 2024 [34]	✓		✓			✓	
Pesantez, 2022 [35]	✓	✓	✓			✓	
Kumar, 2024 [36]	✓		✓			✓	
Ali, 2016 [39]	✓		✓	✓			✓
Sridharan, 2021 [32]	✓			✓			
Gomez, 2021 [9]	✓		✓	✓			
Hu, 2021 [10]	✓		✓	✓			
Kim, 2021 [12]	✓		✓	✓			
**Present Work**	✓	✓	✓	✓	✓		

**Table 2:** Notations used in this article.

Notation	Description
*M*	Total number of smart meters in the system
*N*	Total number of wireless channels available in the system
*T*	Time intervals for smart meter transmissions
*W*	Transmission window length around the preferred interval
*Z*	Set of transmission frames in the system
*F*	Specific transmission frame
TH	System throughput
$N_{r}$	Number of channels reserved for mobile users
$N_{f}$	Number of fixed channels assigned to the Mobile Virtual Operator (MVO)
$N_{ra}$	Number of random channels available for the MVO under certain conditions
$n_{c}$	Number of channels occupied by mobile users in a given interval
$n_{a}$	Number of smart meters scheduled to transmit in an interval
$n_{a}^{*}$	Number of smart meters that effectively transmit in an interval
$t_{k}$	Preferred time interval for smart meter *k*
$x_{h,t,k}$	Binary variable indicating whether meter *k* uses channel *h* in interval *t*
$z_{h,t,k}$	Binary variable indicating whether meter *k* has effectively transmitted in channel *h* and interval *t*
$z_{f,k}$	Indicator of effective transmission in frame *f* for SM *k*, taking value 1 if transmission occurs in *f*
*h*	Channel assigned to a SM
*t*	Time interval
$t_{f}$	Time interval associated with frame *f*
*k*	*k*-th smart meter
$w_{k}$	Fairness weight based on accumulated tokens for SM *k*
$w_{t,k}$	Temporal distance weight between interval *t* and preferred interval $t_{k}$
$w_{h,k}$	Weight depending on the channel chosen for SM *k*
$T_{k}\left(0\right)$	Initial token of meter *k*, starting without accumulated tokens
$T_{k}\left(n+1\right)$	Token increment for SM *k* after a failed attempt
$W(T_{k})$	Transmission weight adjusted according to accumulated tokens $T_{k}$
$w_{F,k}$	Final weight calculated for SM *k* from $w_{k}$, $w_{t,k}$, and $w_{h,k}$
${\bar{T}}_{k}$	Average of accumulated tokens for SM *k* in simulations

**Table 3:** Summary of characteristics of the simulated scenarios.

	Group 1 ($p_{a}=0.001$)					Group 2 ($p_{a}=0.01$)			
#	Name	$p_{a}$	$p_{d}$	$N_{r}$	#	Name	$p_{a}$	$p_{d}$	$N_{r}$
1	Scenario 1	0.001	0.005	0	21	Scenario 21	0.01	0.005	0
2	Scenario 2	0.001	0.005	1	22	Scenario 22	0.01	0.005	1
3	Scenario 3	0.001	0.005	2	23	Scenario 23	0.01	0.005	2
4	Scenario 4	0.001	0.005	3	24	Scenario 24	0.01	0.005	3
5	Scenario 5	0.001	0.005	4	25	Scenario 25	0.01	0.005	4
6	Scenario 6	0.001	0.010	0	26	Scenario 26	0.01	0.010	0
7	Scenario 7	0.001	0.010	1	27	Scenario 27	0.01	0.010	1
8	Scenario 8	0.001	0.010	2	28	Scenario 28	0.01	0.010	2
9	Scenario 9	0.001	0.010	3	29	Scenario 29	0.01	0.010	3
10	Scenario 10	0.001	0.010	4	30	Scenario 30	0.01	0.010	4
11	Scenario 11	0.001	0.025	0	31	Scenario 31	0.01	0.025	0
12	Scenario 12	0.001	0.025	1	32	Scenario 32	0.01	0.025	1
13	Scenario 13	0.001	0.025	2	33	Scenario 33	0.01	0.025	2
14	Scenario 14	0.001	0.025	3	34	Scenario 34	0.01	0.025	3
15	Scenario 15	0.001	0.025	4	35	Scenario 35	0.01	0.025	4
16	Scenario 16	0.001	0.040	0	36	Scenario 36	0.01	0.040	0
17	Scenario 17	0.001	0.040	1	37	Scenario 37	0.01	0.040	1
18	Scenario 18	0.001	0.040	2	38	Scenario 38	0.01	0.040	2
19	Scenario 19	0.001	0.040	3	39	Scenario 39	0.01	0.040	3
20	Scenario 20	0.001	0.040	4	40	Scenario 40	0.01	0.040	4

## Data Availability

Data are contained within the article.
